# Antifungal and Antioxidant Properties of Chitosan Polymers Obtained from Nontraditional *Polybius henslowii* Sources

**DOI:** 10.3390/md17040239

**Published:** 2019-04-22

**Authors:** Francisco Avelelas, André Horta, Luís F.V. Pinto, Sónia Cotrim Marques, Paulo Marques Nunes, Rui Pedrosa, Sérgio Miguel Leandro

**Affiliations:** 1 MARE—Marine and Environmental Sciences Centre, ESTM, Instituto Politécnico de Leiria, 2520-641 Peniche, Portugal; franciscoavelelas@gmail.com (F.A.); paulo.nunes@ipleiria.pt (P.M.N.); rpedrosa@ipleiria.pt (R.P.); 2 MARE—Marine and Environmental Sciences Centre, Instituto Politécnico de Leiria, 2520-641 Peniche, Portugal; andre.horta@ipleiria.pt (A.H.); sonia.cotrim@ipleiria.pt (S.C.M.); 3 BioCeramed, S.A., Rua José Gomes Ferreira nº 1 - Armazém D 2660-360 São Julião do Tojal, Portugal; info@bioceramed.com; 4 CENIMAT/I3N, Departamento de Ciência dos Materiais, Faculdade de Ciências e Tecnologia FCT, Universidade Nova de Lisboa, Campus da Caparica, 2829-516 Caparica, Portugal; 5 Instituto Português do Mar e da Atmosfera (IPMA) Rua Alfredo Magalhães Ramalho, 6, 1449-006 Lisboa, Portugal

**Keywords:** *Polybius henslowii*, marine resources, chitosan, chitooligosaccharides, antifungal activity, antioxidant activity

## Abstract

Chitin was extracted from *Polybius henslowii*, a swimming crab, captured in large quantities throughout the Portuguese coast by purse seine vessels as bycatch. After standard chitin extraction procedures, water-soluble chitosan products were obtained via two different methods: (1) *N*-acetylation with the addition of acetic anhydride and (2) a reaction with hydrogen peroxide. The chemical structure and molecular weight of chitosan derivatives, water-soluble chitosan (WSC) and chitooligosaccharides (COS), were confirmed by Fourier Transform Infrared Spectroscopy (FT-IR) and gel permeation chromatography (GPC). Antioxidant and metal chelation activities were evaluated, and the growth inhibition capacity was tested on four phytopatogens. The chitooligosaccharides from pereopods (pCOS) and shell body parts (sCOS) inhibited all fungal species tested, particularly *Cryphonectria parasitica* with 84.7% and 85.5%, respectively. Both radical scavenging and antifungal activities proved to be dose-dependent. Chitooligosaccharides with a low molecular weight (2.7, 7.4, and 10.4 Kg·mol^−1^) showed the highest activity among all properties tested. These results suggested that chitosan derivatives from *P. henslowii* raw material could potentially be used against phytopathogens or as ingredient in cosmetics and other products related to oxidative stress.

## 1. Introduction

Fungal pathogens are responsible for huge economic losses worldwide. These pathogens may cause damage to roots, crowns, stems, leaves, and fruits of a large range of economically important plants. In Portugal, several fungi species, such as *Phytophthora cinnamomi, Botrytis cinerea, Cryphonectria parasitica*, and *Heterobasidion annosum* cause considerable damage in cork oak forests [1], vegetable crops [2], chestnut trees [3], pines, and conifers [4], respectively. Since chemical fungicides such as sulfur dioxide [5] raise health concerns, the agrochemical industry has been searching for less toxic products. 

Chitosan, a straight-chain polymer of glucosamine and *N*-acetylglucosamine [6] has emerged as a promising antimicrobial material [7]. As they are obtained from chitin present in crustacean’s exoskeleton and fungi cell walls, chitosan products are biocompatible and biodegradable, with a wide range of applications such as in wastewater treatment, food, cosmetics, agrochemicals, cell culture, textiles, and medical devices [8]. Chitosan also exhibits antioxidant activity [9,10] and, therefore, could also be used as a replacement for synthetic antioxidants such as butylated hydroxytoluene (BHT), butylated hydroxy-anisole (BHA), and tert-butylhydroquinone (TBHQ) [11]. 

In recent years, the valorization of fisheries discards by-products has received much attention due to the increasing awareness of its economic potential and environmental impacts [12]. *Polybius henslowii*, a benthopelagic species is found at depths between 0 and 650 m along the eastern Atlantic coasts from Ireland and Britain to the Alborán Sea [13,14] and Morocco [15]. Despite its benthic habit, it also has periodic pelagic phases when larges swarms move along the surface to coastal waters, gathering at high densities from 0 to 14812 individuals per ha [16], with strong interannual oscillations [17]. Although being an extremely abundant marine resource, it is not presently subject to commercial use, and approximately 1240 tonnes/year (mean values from 2004 to 2009) are discarded annually [18]. In Portugal, it is captured as bycatch during the purse seining of *Sardina pilchardus* [18], having a destructive impact on the fishing nets. As such, it is regarded as a plague by the fisherman and not as a potential source of economic incomes. 

The objective of the present study was to determine if this bycatch could be converted into a value-added product by chitin extraction and chitosan production. The products were characterized by gel permeation chromatography (GPC) and FT-IR, and their antioxidant, metal chelation, and antifungal properties were evaluated. 

## 2. Results and Discussion

### 2.1. Polybius henslowii Characterization

The biochemical composition in terms of protein, minerals, lipids, and chitin of the swimming crab *Polybius henslowii* exoskeleton is shown in Table 1.

The shell samples showed a higher percentage of protein and lipids than pereopods. The ash percentage, directly correlated with the calcium carbonate in the exoskeleton, was similar in both body parts. The protein and ash results are in accordance with those reported in previous studies [19], suggesting a higher yield of ash than protein, lipids, or chitin from the crab samples (about 16.6% for protein and 66.6% for ash content). The protein values are in line with the literature [20] for Alaska king crab (from 16.3 to 20.7%) but are lower than those reported for other crab species such as the *Metacarcinus magister* [21], *Callinectes sapidus* [22], and *Carcinus maenas* [23]. Previous studies [23] suggested that differences in the diet of crab at the harvesting site influences their biochemical composition, which could contribute to differences reported in the protein and lipid contents.

Previously published studies on crab species reported values of chitin from 14 to 28% of their total dry weight [24]. The pereopods of the swimming crab showed a higher chitin content than the shells. The chitin values shown in Table 1 are lower than those from other published studies [19,25]; however, authors [25] suggested that differences in chitin yield could be related to such factors as harvest year and/or shell storage duration, which could explain the low yield.

### 2.2. Chitin Extraction Optimization

The conventional demineralization process of crustacean waste uses strong acids such as hydrochloric acid (HCl). This chemical treatment can result in changes to the physiochemical properties of chitin (the hydrolysis of the chitin chains that reduces the average molecular weight of the biopolymer), produces harmful effluent wastewater, and contributes to the cost of the chitin purification process [26]. A mineral-free chitin with a very low ash content is usually required for applications with a very low impurity tolerance, such as biomedical and nutrition products [8]. Therefore, three different HCl concentrations were tested to minimize chitin chain damage and acid use. 

The mineral content in pereopods and shell samples after treatment with HCl was different regarding calcium carbonate removal (Table 2). The lowest ash content was found for the pereopods and shells after treatment with 1 M HCl (Table 2). Nevertheless, all treatments (0.5, 0.75, and 1 M) promoted a high ash removal. 

For an efficient chitin extraction, the associated proteins should be removed at a second stage. Deproteinization carried out with sodium hydroxide (NaOH) at elevated temperatures promoted the removal of protein from crab wastes. Residual protein was determined after each wash in order to evaluate the efficiency of each concentration applied. After treatment with NaOH, the protein values were highest in shells and pereopods treated with 0.5 M NaOH. The residual protein concentration was higher when 0.5 and 0.75 M of NaOH were used, rather than 1 M NaOH (Table 2). 

A high-quality chitosan product should have less than 1% protein [27]. Moreover, a complete removal of protein is desirable, since it allows a higher solubility of chitosan after deacetylation [28]. 

### 2.3. Chitosan and Water-Soluble Chitosan Characterization

The data for chitosan samples characterization in terms of yield, dynamic viscosity, degree of deacetylation, and molecular weight are shown in Table 3. The degree of deacetylation (DD) of chitosan products was determined by infrared spectroscopy analysis. The ratio A_1320_/A_1420_ was slightly different between the samples. The degree of deacetylation of chitosan obtained from shell chitin was 95.1 ± 0.01%, while the samples from pereopods were 91 ± 0.04% deacetylated. The difference is probably due to the longer deacetylation process of 7 h, the time needed to allow a complete dissolution in 1% acetic acid. Therefore, chitosan from pereopods resulted in a higher M_w_ (378 kg.mol^−1^) and dynamic viscosity (749.2 ± 62.7 cP). Since the average M_w_ and dynamic viscosity are closely related, the decrease in these values is consistent with a prolonged reaction time. Previous work [29] demonstrated that a longer exposure to NaOH during deacetylation resulted in a decrease in M_w_ and dynamic viscosity. The yield of chitosan as a percentage of the crab dry weight from both body parts also showed lower values when compared with those from other studies [30], a consequence of the small amount of chitin extracted from the initial raw material.

Chitosan is only soluble in acidic solutions, which limits its applications. The solution viscosity is usually also quite high, which makes it difficult to prepare highly concentrated solutions. In contrast, chitooligosaccharides and other water-soluble chitosan derivatives allow highly concentration solution with low viscosities [31].

In the present study, hydrogen peroxide proved to be an efficient tool for chitosan degradation due to the formation of reactive hydroxyl radicals by the dissociation of hydrogen peroxide [32]. The results in Table 3 show the production of chitooligosaccharides (COS) from both segmented body parts with 7.4 kg.mol^−1^ for pCOS and 2.7 kg.mol^−1^ for sCOS, as well as for commercial chitosan chitooligosaccharides (ccCOS) with 10.4 ± 0.7 kg.mol^−1^. Due to the decrease in molecular weight and degree of deacetylation, these products showed a good solubility in distilled water as previously observed by other authors [33]. 

Besides COS, another derivative soluble in water (WSC) was prepared through the *N*-acetylation of degraded chitosan. The water-soluble chitosan (WSC) products had a molecular weight range of 378 to 404 kg.mol^−1^ in pWSC, of 247 to 279 kg.mol^−1^ in sWSC, and of 780.0 to 775 Kg.mol^−1^ in ccWSC. According to previous studies [34], the reason for the increased solubility of chitosan was the destruction of intramacromolecular and interchain hydrogen bonds, which alters the secondary structure of chitosan, decreasing its crystallinity and unfolding its molecular chains. Also, the degree of deacetylation decreased likely due to the acetylation reaction induced by the acetic anhydride acting as a source of acetyl group for the amines.

### 2.4. DPPH Radical Scavenging Activity

One important mechanism of antioxidation involves scavenging of hydrogen radicals. DPPH (2,2-diphenyl-1-picrylhydrazyl) has a hydrogen-free radical with a characteristic absorption at 517 nm, allowing its detection as the purple color of the DPPH solution fades rapidly when it reacts with proton radical scavengers [35].

**Figure 1:**
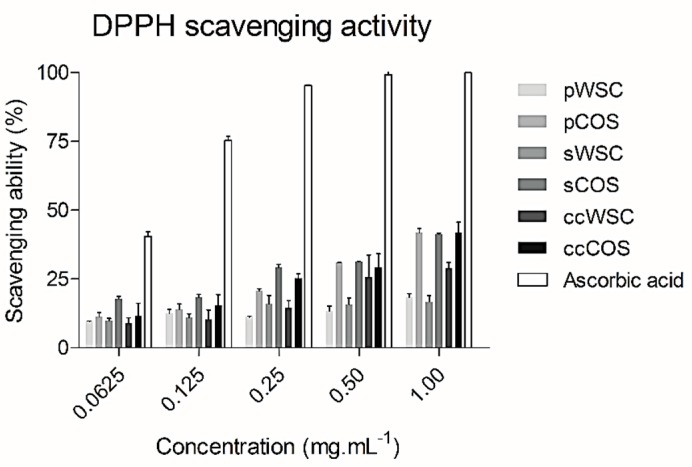
The scavenging ability of water-soluble chitosan (WSC), chitooligosaccharides (COS), and ascorbic acid on 1,1-diphenyl-2-picrylhydrazyl radicals: The values are means of eight replicates ± standard errors.

The DPPH radical scavenging activity of WSC, COS, and ascorbic acid is shown in Figure 1. At 1 mg·mL^−1^, all chitosan products exhibited the highest scavenging ability. The scavenging ability proved to be dose-dependent. 

pCOS, sCOS, and ccCOS showed the highest scavenging activity. The WSC products showed a lower activity than COS at all concentrations tested (Figure 1). Also, no differences were noted between products obtained from *P. henslowii* and the commercial product. However, compared to the scavenging ability of ascorbic acid, all products had lower values.

Previous studies proved that the antioxidant activity of chitosan depends on the molecular weight and the degree of deacetylation [36,37].

Since chitosan chains have active hydroxyl and amino groups that can react with free radicals [38], the scavenging activity of chitosan may be due to the reaction between the free radicals and protonated amino groups [38]. Several researchers suggested that the scavenging mechanism of chitosan was based on the reaction of hydroxyl and superoxide anion radicals with active hydrogen atoms in chitosan to form a stable macromolecule radical. In the chitosan structure, there are three hydrogen sources, at the C−2 (NH_2_), C−3 (OH) and C−6 (OH) positions respectively [39]. The present results support this theory, suggesting that the number of free amino groups is definitely important to a good antioxidant performance, since a high degree of deacetylation resulted in chitosans (COS) with better antioxidant properties. Other studies revealed the contribution of a prolonged *N*-deacetylation and its impact on the scavenging activities through the production of highly deacetylated products [40,41]. Once again, this seems to be in line with our findings, when compared the chitooligosaccharides (DD: 86−93%) and water-soluble chitosan (DD: 55−62%), proving that amino groups are a possible major factor for free radical scavenging activity.

In addition, previous investigations have revealed that the DPPH radical scavenging activity of chitosan increased with a decreasing molecular weight (M_W_) [36,41]. According to previous studies [36], a high-M_W_ chitosan (WSC) would have a lower mobility than a low-M_W_ chitosan (COS). Consequently, this would increase the possibility of inter- and intramolecular bonding of the high-M_W_ chitosan molecules; thus, the chance of exposure of their amine groups might be restricted. An approximate 20% scavenging ability with 1 mg·mL^−1^ of chitosan from crustaceans has been reported previously [41].

The results obtained in the current study suggest that the degradation of chitosan by hydrogen peroxide enhanced not only the solubility but also its antioxidant activity, supporting the use of *P. henslowii* as raw material for the manufacturing of chitosan products towards antioxidant applications.

### 2.5. Superoxide Radical (O_2_^−^) Scavenging Ability

Superoxides are radicals of which unpaired electrons are on oxygen. Despite their limited chemical reactivity, they can form more dangerous species, including singlet oxygen, hydrogen peroxide, and hydroxyl radicals in living organisms [42,43]. Further, superoxides were also known to indirectly initiate lipid peroxidation as a result of H_2_O_2_ formation, creating precursors of hydroxyl radicals [44]. 

In the present study a superoxide radical scavenging assay was based on the capacity of water-soluble chitosan (WSC) and chitooligosaccharides (COS) to inhibit the reduction of nitro blue tetrazolium (NBT).

**Figure 2:**
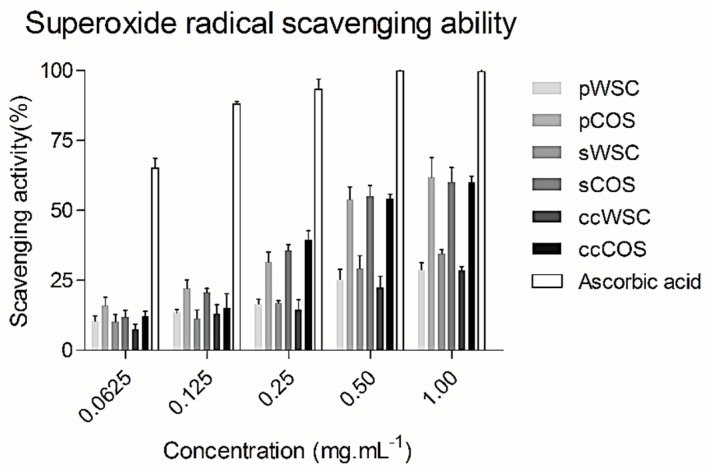
The scavenging ability of water-soluble chitosan (WSC), chitooligosaccharides (COS), and ascorbic acid on superoxide radical: The values are means of eight replicates ± standard errors.

Figure 2 summarizes the scavenging effects of WSCs and COSs produced from *P. henslowii* raw material (pereopods and shells) and commercial chitosan on superoxide radicals within a concentration range from 0.0625 to 1 mg·mL^−1^. All the products scavenged superoxide in a concentration-dependent manner. The figure showed that chitooligosaccharides (COSs) had the highest scavenging activity towards superoxide anion radicals with concentrations above 0.25 mg·mL^−1^. Again, no differences were reported between products obtained from *P. henslowii* and commercial samples for both WSCs and COSs. At 0.0625 mg·mL^−1^, all samples proved a low scavenging activity with values changing from 9.2 ± 1.9% (ccWSC) to 16.1 ± 2.9% (pCOS). On the other hand, EDTA proved much higher superoxide scavenging values (65.1 ± 3.6%) within the same concentrations (0.0625 mg·mL^−1^). While testing the highest concentration at 1 mg·mL^−1^, the pCOS, sCOS, and ccCOS samples clearly exhibited a higher scavenging activity (61.9 ± 7%, 60.1 ± 5.2%, and 57.5 ± 4.1%, respectively) when compared to pWSC, sWSC, and ccWSC (28.7 ± 2.7%, 31.9 ± 4.5%, and 28.5 ± 1.5%, respectively)

Previously published studies [45,46,47] suggested a relationship between molecular weight and the ability to scavenge superoxide anions. Compared with chitosan, chitooligosaccharides have very short chains and the ability to form intramolecular hydrogen bonds (O_3_–O_5_ and N_2_–O_6_) decreases, which means that the hydroxyl and amino groups are activated, being helpful to the reaction with superoxide anions. This fact may be related once again to the formation of strong intermolecular and intramolecular hydrogen bonds that reduced the reactivity of hydroxyl and amino groups in the polymer chains. Other authors [48] proved the influence of hydroxyl and amino groups in the scavenging process, showing lower scavenging values for chitosan-thiamine pyrophosphate (CS-TPP) and chitosan-hydroxybenzotriazole (CS-HOBt) when compared to chitosan-acetate and chitosan-EDTA. According to the author, it might be due to the higher ability of TPP and HOBt to bond with the hydroxyl and amino groups of chitosan, blocking the reaction with the superoxide.

### 2.6. Chelating Ability on Ferrous Ions

The generation of radicals can be retarded by chelation of ferrous ions [49], being chitosan and chitosan derivatives reported as significant chelators [50]. This is why chitosan can be considered as a potential natural antioxidant to prolong food shelf life [40].

**Figure 3:**
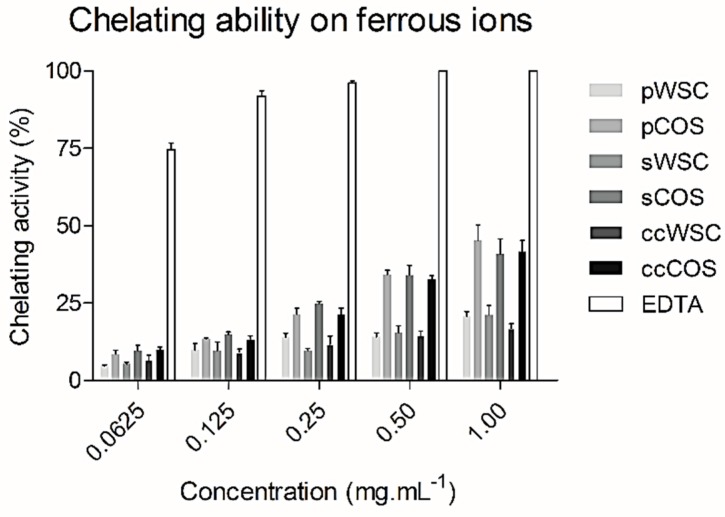
The chelating ability of water-soluble chitosan (WSC), chitooligosaccharides (COS), and ethylenediaminetetraacetic acid (EDTA) on ferrous ions: The values are means of eight replicates ± standard errors.

Figure 3 shows the ferrous-ion chelating ability of WSCs and COSs produced from *P. henslowii* raw material (pereopodes and shells) and from commercial chitosan within a concentration range from 0.0625 to 1 mg·mL^−1^.

In general, the chelation ability of all WSCs with concentrations ranging from 0.0625 to 1 mg·mL^−1^ was relatively low. On the contrary, EDTA exhibited an excellent ferrous ion-chelating capacity of approximately 74.6 ± 4.1% at a concentration of 0.0625 mg·mL^−1^. Nevertheless, all products proved a high chelation ability within 1 mg·mL^−1^, showing COSs the highest activities, from 40.7 ± 10.1% (sCOS) to 45.2 ± 10% (pCOS). When tested at 0.5 and 1 mg·mL^−1^, COSs almost presented a two-times-higher chelating power than WCSs. Also, no differences were seen between sCOS, pCOS, and ccCOS. Still, EDTA proved to be much more potent at 1 mg·mL^−1^, chelating all ferrous ions (100%). Other authors [51] reported similar activities for EDTA when compared to the N-alkylated disaccharide chitosan derivative. Despite the close relation of the chitosan metal ion absorption capability to its amino acid content and their distribution, other factors such as affinity for water, pH, temperature, and crystallinity also affected the ion-chelating activity [52,53].

In addition, the ion-chelating activity of chitosan seems to be strongly affected by the degree of acetylation, with the fully acetylated chitosan showing very little chelating activity [54]. Similar findings were reported in the present study, revealing higher degrees of deacetylation (COSs) and a stronger effect on metal chelation abilities, when compared to low deacetylated products (WSCs). 

### 2.7. Antifungal Assay

**Figure 4:**
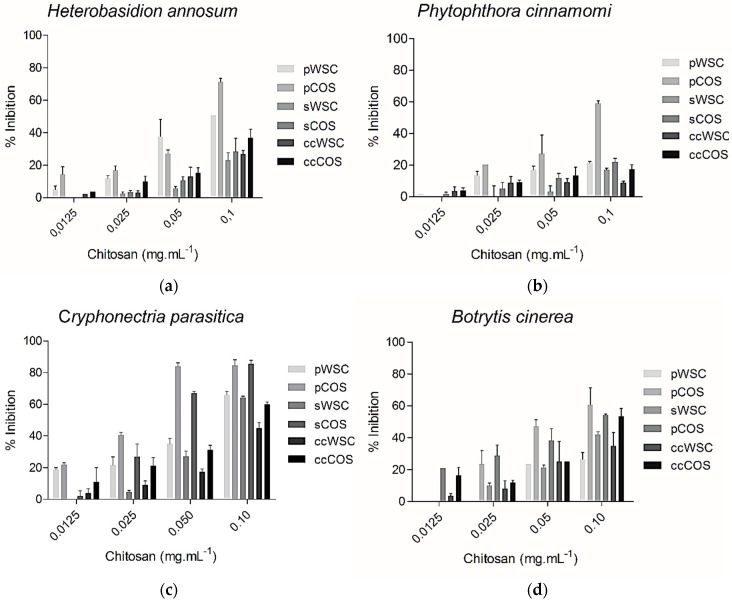
The effect of COS and WSC products (concentration ranging from 0.0125 to 0.1 mg·mL^−1^) on the growth of *Heterobasidion annosum* (**a**), *Phytophthora cinnamomi* (**b**), *Cryphonectria parasitica* (**c**), and *Botrytis cinerea* (**d**): The values are means of eight replicates ± standard error.

The molecular weight of chitosan is known to influence its ability to inhibit the activity of several fungal species [55]. The antifungal activity of WSC and COS products towards the plant pathogens *B. cinerea*, *H. annosum*, *P. cinnamomi*, and *C. parasitica* is shown in Figure 4. The concentrations of chitosan ranged from 0.0125 to 0.1 mg·mL^−1^. The antifungal activity of all chitosan products proved to be dose-dependent, but despite the observed inhibitory effect, none of the tested fungal species were completely inhibited by COS or WSC at the highest concentrations. However, pCOS and sCOS showed a good inhibition against *Cryphonectria parasitica.* Overall, pCOS exhibited a higher activity against all other tested species than sCOS, pWSC, and sWSC.

Furthermore, the results showed that the capability to inhibit fungal growth was clearly higher for COS from both segmented body parts, especially the pereopods, than for WSC products. While *P. cinnamomi* exhibited a lower vulnerability towards all chitosans, *B. cinerea* and *C. parasitica* were highly inhibited by COS. *B. cinerea* sensitivity to chitosan has been reported previously [56].

Several mechanisms have been proposed for the antimicrobial action of chitosan. It has been suggested that chitosan may inhibit microbial growth by acting as a chelating agent rendering metals, trace elements, or essential nutrients unavailable for the organism to grow at a normal rate [57]. This could be caused by the accumulation of chitosan precipitates on the membrane surface, as the physiological pH in microbial cells is around neutral [58,59]. On the contrary, water soluble chitosan derivatives, due to their solubility in neutral solutions, would be unable to form such a layer, and therefore, no antimicrobial activity would be expected. 

Another hypothesis explains the activity of chitosan as being based on the electrostatic interaction of the protonated amino groups with the negatively charged cell wall surface of the targeted microorganisms, which can lead to the disruption of the cell wall and, therefore, to its death [60]. The importance of protonation has been reported by several studies [61,62,63], which proved that only positively charged chitosan’s are able to inhibit microbial growth.

Moreover, the degree of deacetylation certainly plays an important role not only on the antioxidant activity but also in antifungal activity, since the products with a higher degree of deacetylation (COS), exhibited a higher growth inhibition. Again, the number of free amino groups seems to influence bioactivities. Also, it is possible that chitosan’s antifungal activity is caused by its chains, which interact with the cell’s membrane and inhibits the intracellular functions [64]. Recently published studies [65] suggested that interactions between low molecular weight chitosans, DNA, RNA, and protein could partly explain the effects of chitosan on translation efficiency. In addition, other studies showed that the effectiveness of chitosan did not depend solely on the chitosan formulation but also on the *type* of fungi [61]. This is in accordance with our findings, since both pathogens from the phylum Ascomycota (*Botrytis cinerea* and *Cryphonectria parasitica*) were highly inhibited at 0.1 mg·mL^−1^ when compared to the remaining species, *Heterobasidion annosum* (phylum Basidiomycota) and *Phytophthora cinnamomi* (phylum Heterokontophyta).

## 3. Experimental Section

### 3.1. Biochemical Characterization of Raw Materials

Swimming crab, *Polybius henslowii* was by-catched by fishing vessels when capturing *Sardina pilchardus* along the west coast of Peniche (39°24´22.19´´N, 9°35´50.51´´O), Portugal. Dead organisms were first boiled, then dried in an oven at 100 °C for 2 days, and segmented into shell and pereopods. The raw material was powdered into particles with diameters ranging from 150 to 500 μm. The ash content was determined by initially drying the raw material in an oven at 100 °C for 6h. The dried samples were placed in a furnace at 530 °C for 20 h, and the remaining material was weighed after cooling in a desiccator. The microbiuret method was used for protein assays [66], and the values were compared with a standard curve established with known concentrations of bovine serum albumin (BSA). Free fat was extracted from the raw material by Soxhlet using ether as the solvent [67]. Three replicates were conducted for each sample.

### 3.2. Chitin Extraction and Deacetylation

Chitin was isolated according to Reference [19] with some modifications. Demineralization was carried out with three different concentrations of HCl in order to optimize the extraction of calcium carbonate: 0.5, 0.75, and 1 M at 21°C for 30 min (ratio of 1:30, *w/v*). After washing with distilled water and drying in an incubator for 24 h at 40 °C, the samples were subjected to different concentrations of sodium hydroxide (0.5 M, 0.75 M, and 1 M of NaOH; ratio of 1:15, *w/v*), thereby removing the organic matter. The shells and pereopods were incubated at 70 °C for 2 h in a water bath and washed with distilled water until a neutral pH was achieved. The demineralization and deproteinization efficiencies were respectively determined through the ash content and microbiuret assay measurements.

The chemical deacetylation of chitin was achieved by subjecting chitin to 12 M of NaOH solution at 120 °C for 3 h (pereopods) and 7 h (shells). After the alkali treatment, chitosan was collected and washed with distilled water to remove NaOH residues until a neutral pH was reached. These conditions yielded chitosan products from both body parts, which exhibited a complete dissolution in acetic acid (1%, *v/v*) at 25 °C.

Three batches were performed in each step, therefore having a minimum of three replicates per sample.

### 3.3. Chitooligosaccharides and Water-Soluble Chitosan Production

Water-soluble chitosan (WSC) was prepared according to a previously published procedure [34], through *N*-acetylation with acetic anhydride (Ac_2_O). One gram of chitosan was dissolved in 25 mL of 2.8 % acetic acid, and 25 mL of ethanol were added. Acetic anhydride was charged after a complete dissolution of chitosan and left shaking for 4 h. The reaction mixture was precipitated with ethanol and dried at 55 °C for 24 h.

The production of chitooligosaccharides (COS) was derived by adding hydrogen peroxide (H_2_O_2_) [32]. One gram of chitosan was dissolved in 20 mL of 2% (*w/w*) acetic acid. After a complete dissolution, 5.5% H_2_O_2_ was added to the mixture and incubated at 37 °C for 4 h. Then, chitooligosaccharide products were precipitated with ethanol and dried at 55 °C for 24 h. The same procedure was carried out with the commercial chitosan (cc) product (Altakitin S.A., Portugal) in order to compare its physical and biochemical properties. All samples were performed in triplicate.

### 3.4. Chitosan Product Characterization

The dynamic viscosity was determined in triplicate with a rotational Haake viscotester 7 plus at (20 ± 1) °C after the samples were dissolved in 1% (*v/v*) acetic acid. The molecular weight (M_w_) of chitosan products was determined by gel permeation chromatography (GPC) [68] with some variations. A Varian PL aquagel-OH MIXED bed column (Varian, France) 8-μm column was used. The samples (approx. 5 mg) were dissolved in 1 mL of eluent (AcOH/AcONa buffer pH 4.5, Panreac Spain) and filtered through a microsyringe filter prior to injection. The flow rate was 1 mL/min. A calibration curve was obtained by using Varian pullulan polysaccharides certified standards (Varian, France) at the same chromatographic conditions. FTIR spectroscopy (Bruker FTIR-ATR spectrophotometer) was employed to determine the degree of acetylation (DA) [69]. The spectra of all samples were recorded in KBr pellets (Sigma Aldrich, Steinheim, Germany) by the accumulation a minimum of 64 scans with a resolution of 4 cm^−1^.

### 3.5. Scavenging of 1,1-diphenyl-2-picrylhydrazyl Radicals 

The chitosan scavenging of 1,1-diphenyl-2-picrylhydrazyl radicals (DPPH) was determined according to a previously described method [70]. DPPH (Sigma Aldrich, Steinheim, Germany) solution was prepared at 0.1 mM with methanol. A volume of 10 µL of each sample was added to 990 μL of a DPPH solution. The sample concentrations varied from 0.0625 to 1 mg·mL^−1^. The reaction mixture was shaken vigorously and stored in the dark at room temperature for 30 min. The absorbance was read at 517 nm in a microplate reader (Biotek, Vermont, USA). All samples were run in triplicate. Ascorbic acid was used for comparison. The free radical scavenging activity was calculated by the following Equation (1):

Scavenging activity (%) = (1 − Abs_sample_/Abs_control_) × 100 (1)

### 3.6. Superoxide Radical (O_2_^−^) Scavenging Activity

The superoxide scavenging ability of all water-soluble chitosan (WSC) and chitooligosaccharide (COS) samples was assessed by the method of Reference [71]. This assay was based on the reduction of nitro blue tetrazolium (NBT) in the presence of NADH and phenazine methosulphate (PMS) under aerobic condition. The 3.00 mL reaction mixture contained 50 µL of lM NBT, 150 µL of l M nicotinamide adenine dinucleotide (NADH), and Trisbuffer (0.02 M, pH 8.0). The samples were added with concentrations ranging from 0.0625 to 1.0 mg·mL^−1^. The reaction started by adding 15 µL of lM phenazine methosulfate (PMS). After incubation at room temperature for 5 min, 300 µL of each reaction were transferred to a 96-well plate, and the absorbance was recorded at 560 nm against a blank in microplate reader (Biotek, Vermont, USA). EDTA was used as a positive control. The capability of scavenging to superoxide radical was calculated using the following Equation (2):

Scavenging effect (%) = (1 − A_sample_/A_control_) *×* 100 (2)

### 3.7. Chelating Ability on Ferrous Ions

The ferrous ion-chelating potential of all chitosan products (WSC and COS) was investigated according to the method of Decker and Welch [72]. Each WSC and COS sample (0.0625–1.0 mg·mL^−1^) in a 0.2% acetic acid solution was mixed with 0.1 mL of FeCl_2_ (2 mM) and 3.7 mL of methanol. The reaction was initiated by adding 2.0 mL ferrozine (5 mM), shaken vigorously and incubated for 10 min in the dark at room temperature. The ferrous ion-chelating ability was determined by the absorbance at 562 nm against a blank in a microplate reader (Biotek, Vermont, USA). EDTA was used as a positive control. The ability of WSC and COS to chelate ferrous ions was calculated according to the following Equation (3):

Chelating activity (%) = (1 − Abs_sample_/Abs_control_) × 100 (3)

### 3.8. Effect of Chitosan on Mycelial Growth

*Cryphonectria parasitica* (DSMZ 62626)*, Phytophthora cinnamomi* (DSMZ 62654), *Botrytis cinerea* (DSMZ 4709), and *Heterobasidion annosum* (DSMZ 1531) were all purchased from the Leibniz Institut DSMZ (German Collection of Microorganisms and Cells Cultures), Germany. The four strains were grown for 5 days at 28 °C on potato dextrose agar (PDA) plates. The antifungal assessment of the chitosan products was conducted through the mycelial radial growth inhibition technique on a PDA medium according to El Ghaouth et al. [73]. Mycelial discs 4 mm in diameter were cut from the margins of the initial colony and placed on PDA plates containing different concentrations of chitosan, ranging from 0.0125 to 0.1 mg·mL^−1^. Control plates of PDA were prepared without chitosan. The PDA plates were incubated at (23 ± 2) °C for 7 days. The mycelial radial growth was measured when the control colony had grown to the edge of the plate. The diameter of each fungal colony prepared in triplicate was measured in mm. and the activity was expressed as the inhibition of mycelial growth.

## 4. Conclusions

This study shows that *P. henslowii* captured as bycatch could be applied as a source of chitosan derivatives. The swimming crab characterization clearly showed that, considering its composition, different segments of its body could be applied to several biotechnological applications such as feed supplements, adding value to the raw material. Moreover, the bioavailability of this marine resource, the optimization of chemical processes (through solvent reuse), and, considering all production costs, the final price could be highly competitive compared to the current market offer. The results suggested that the chitosan derivatives, mainly chitooligosaccharides obtained through processing of *P. henslowii* raw material, could be effectively employed as an ingredient in cosmetics or functional products in order to decrease oxidative stress. In general, the chitosan derivatives produced from the raw material of *P. henslowii* remarkably inhibited the tested phytopathogenic fungi, mainly *Cryphonectria parasitica*. In addition, these samples produced an interesting inhibitory activity when compared with commercial chitosan derivatives. However, in order to promote the use of this raw material, extensive research on its characterization (RNM) and other applications (antibacterial activity and allergenic tests) must be addressed.

## Figures and Tables

**Table 1:** The characterization of dried *P. henslowii* expressed as a percentage of dried weight (% of DW). Mean value (± SD).

Raw Material	Protein (%)	Ash (%)	Lipids (%)	Chitin (%)
Shell	32.1 ± 6.68	44.5 ± 0.57	13.2 ± 0.25	9.7 ± 0.57
Pereopods	16.6 ± 1.21	49.3 ± 5.86	1.6 ± 0.14	11.4 ± 0.19

**Table 2:** The ash and protein contents of segmented body parts of *P. henslowii* after treatment with 1 M, 0.75 M, and 0.5 M HCl and NaOH. Mean value (± SD).

**NaOH/HCl**	**Shells Samples**			
	**Protein Content (%)**	**Protein Removal (%)**	**Ash Content (%)**	**Ash Removal (%)**
**1 M**	2.0 ± 0.12	96.1 ± 0.25	0.8 ± 0.01	98.2 ± 0.02
**0.75 M**	2.3 ± 0.14	95.35 ± 0.28	1.0 ± 0.05	97.8 ± 0.11
**0.5 M**	2.29 ± 0.15	95.39 ± 0.31	1.2 ± 0.17	97.3 ± 0.38
**NaOH/HCl**	**Pereopods Samples**			
	**Protein Content (%)**	**Protein Removal (%)**	**Ash Content (%)**	**Ash Removal (%)**
**1 M**	1.2 ± 0.12	92.2 ± 0.78	0.4 ± 0.19	99.1 ± 0.4
**0.75 M**	1.5 ± 0.07	90.5 ± 0.48	0.5 ± 0.01	98.9 ± 0.8
**0.5 M**	1.8 ± 0.06	88.2 ± 0.43	0.7 ± 0.05	98.6 ± 0.1

**Table 3:** Chitosan yield (%), dynamic viscosity (cP), deacetylation degree (DD%), and molecular weight (kg.mol^−1^) obtained from *Polybius henslowii* raw material. pWSC—Pereopods water-soluble chitosan; pCOS—pereopods chitooligosaccharides; sWSC—shells water-soluble chitosan; sCOS—shells chitooligosaccharides. Mean value (± SD).

Chitosan Products	Yield (%)	Dynamic Viscosity (cP)	DD (%)	M_w_ (kg·mol^−1^)
Pereopods chitosan	9.7 ± 0.62	749.2 ± 62.69	94.3 ± 0.04	378.2 ± 78.00
pWSC	-	-	62 ± 0.53	404.0 ± 45.00
pCOS	-	-	93.3 ± 0.04	7.4 ± 1.20
Shells chitosan	8.0 ± 0.24	417.2 ± 94.99	95.1 ± 0.01	247.0 ± 31.20
sWSC	-	-	55.0 ± 3.21	279.0 ± 33.00
sCOS	-	-	95.0 ± 0.62	2.7 ± 0.40
Commercial chitosan	-	-	87.0	780.0
ccWSC	-	-	57.0 ± 0.83	775.0 ± 42.00
ccCOS	-	-	86.0 ± 1.4	10.4 ± 0.70
